# Effects of Probiotic Supplementation on Inflammatory Markers and Glucose Homeostasis in Adults With Type 2 Diabetes Mellitus: A Systematic Review and Meta-Analysis

**DOI:** 10.3389/fphar.2021.770861

**Published:** 2021-12-10

**Authors:** Li-Na Ding, Wen-Yu Ding, Jie Ning, Yao Wang, Yan Yan, Zhi-Bin Wang

**Affiliations:** ^1^ Endocrine and Metabolic Diseases Hospital of Shandong First Medical University, Shandong First Medical University and Shandong Academy of Medical Sciences, Jinan, China; ^2^ Shandong Institute of Endocrine and Metabolic Diseases, Jinan, China; ^3^ Department of Surgery, Qilu Hospital of Shandong University, Jinan, China

**Keywords:** probiotic, inflammation, type 2 diabetes mellitus, gut microbiota, meta-analysis

## Abstract

**Background:** Several studies have revealed the effect of probiotic supplementation in patients with type 2 diabetes (T2DM) on the amelioration of low-grade inflammation, which plays an important role in the pathogenesis of T2DM. However, the effects of the clinical application of probiotics on inflammation in individuals with T2DM remain inconsistent. This study aims to investigate the comprehensive effects of probiotics on inflammatory markers in adults with T2DM.

**Methods:** PubMed, Embase, Cochrane Library, and the Web of Science were searched to identify randomized controlled trials (RCTs) exploring the effect of probiotic supplementation on inflammatory markers in individuals with T2DM through March 11, 2021. Two reviewers independently screened the literature, extracted data, and assessed the risk of bias of the included studies. We used a random-effects model to calculate the standardized mean difference (SMD) between the probiotic supplementation and control groups.

**Results:** Seventeen eligible studies were selected with a total of 836 participants, including 423 participants in probiotic supplementation groups and 413 participants in control groups. Our study demonstrated that compared with the control condition, probiotic intake produced a beneficial effect in reducing the levels of plasma inflammation markers, including tumour necrosis factor-α (TNF-α) (SMD [95% CI]; −0.37 [−0.56, −0.19], *p* < 0.0001) and C-reactive protein (CRP) (SMD [95% CI]; −0.21 [−0.42, −0.01], *p* = 0.040), while it had no effect on the plasma interleukin-6 (IL-6) level (SMD [95% CI]; −0.07 [−0.27, 0.13], *p* = 0.520). In addition, our results support the notion that probiotic supplementation improves glycaemic control, as evidenced by a significant reduction in fasting blood glucose (FPG), HbA1c and HOMA-IR (SMD [95% CI]: −0.24 [−0.42, −0.05], *p* = 0.010; −0.19 [−0.37, −0.00], *p* = 0.040; −0.36 [−0.62, −0.10], *p* = 0.006, respectively).

**Conclusion:** Our study revealed some beneficial effects of probiotic supplementation on improving inflammatory markers and glucose homeostasis in individuals with T2DM. Probiotics might be a potential adjuvant therapeutic approach for T2DM.

**Systematic Review Registration**: [https://www.crd.york.ac.uk/prospero/], identifier [CRD42021235998].

## Introduction

Type 2 diabetes mellitus (T2DM) is one of the most common endocrine and metabolic diseases and is characterized by impaired pancreatic islet β cell function and insulin resistance in insulin target tissues (Gurung et al., 2020; Arora et al., 2021). Data have shown that the incidence of T2DM has been sharply increasing worldwide and has become one of the most critical public health concerns (Zhang and Gregg, 2017). Moreover, due to diabetes-related complications such as blindness, kidney failure, heart attacks, stroke, and lower-limb amputation, T2DM is a leading cause of morbidity and mortality worldwide (Pearson, 2019).

Currently, there is no simple or effective intervention available to prevent and manage diabetes due to the complex and multifactorial nature of T2DM (Pearson, 2019). In recent years, emerging evidence suggests that the gut microbiota is closely related to the development and progression of T2DM (Tsai et al., 2019; Gurung et al., 2020). Some studies have found that the occurrence of T2DM is associated with gut microbial dysbiosis, which plays a major role in promoting chronic low-grade inflammation and insulin resistance and increases the risk of diabetes (Qin et al., 2012; Karlsson et al., 2013; Harkins et al., 2020). A variety of mechanisms have been proposed to explain the role of the gut microbiota in the onset of T2DM, including alteration of the host’s energy homeostasis, intestinal barrier integrity, gastrointestinal peptide hormone secretion, immune response, and inflammatory status (Dongarra et al., 2013; Marchesi et al., 2016; Corb Aron et al., 2021).

**FIGURE 1:**
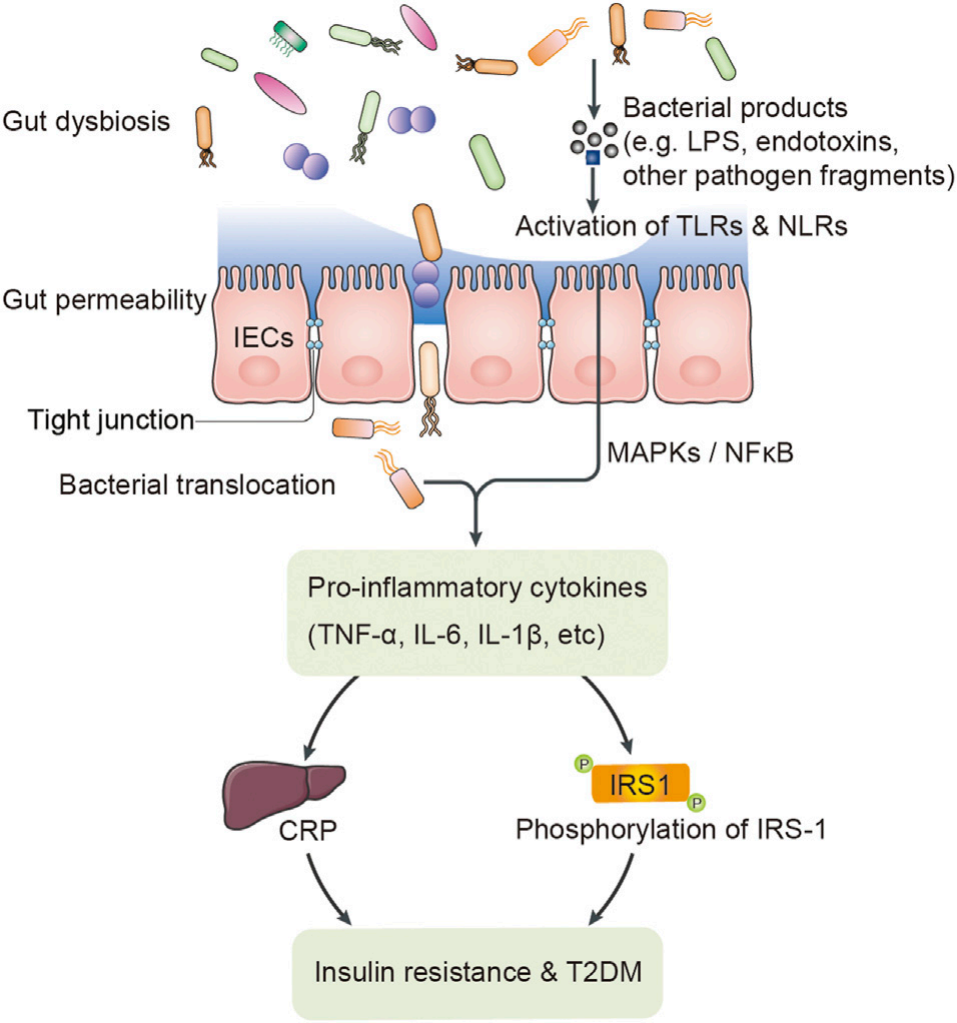
The putative relationship between gut dysbiosis and T2DM.

Several studies have proposed that T2DM is associated with long-term chronic low-grade inflammation characterized by an increase in circulating levels of pro-inflammatory cytokines and chemokines, such as tumour necrosis factor-α (TNF-α) and interleukin-6 (IL-6) (Coope et al., 2016; Eguchi and Nagai, 2017). Elevated levels of TNF-α and IL-6 could also trigger the synthesis of C-reactive protein (CRP) in the liver, which is a common systemic inflammatory biomarker and invariably correlates with insulin resistance and the pathogenesis of T2DM (Badawi et al., 2010; Lainampetch et al., 2019). These pro-inflammatory factors could interfere with the insulin signalling cascade and impair insulin sensitivity by increasing the serine/threonine phosphorylation ratio of insulin receptor substrate 1 (IRS-1) in insulin target tissues (Chen et al., 2015). Pro-inflammatory signalling can also affect pancreatic β-cell functions and impair insulin secretion. In addition, pro-inflammatory factors disrupt endothelial cell functions and limit insulin access to its target tissues, which also contribute to insulin resistance (Figure 1) (Cani et al., 2019; Harkins et al., 2020).

Probiotics play a pivotal role in maintaining the balance of the gut microbiota (Bagarolli et al., 2017; Ardeshirlarijani et al., 2019). Currently, as a potential source of new therapeutics that work by regulating the gut microbiota, probiotics are commonly used to prevent and improve diabetes by altering the gut microbiota and its metabolites (Cani, 2018; Gurung et al., 2020). Among these changes, changes in short-chain fatty acids (SCFAs) and bile acids can improve glucose homeostasis, alleviate insulin resistance, and increase adipose tissue thermogenesis (Descamps et al., 2019; Woldeamlak et al., 2019; Tilg et al., 2020). Interestingly, recent studies have also shown that probiotic supplementation can prevent and improve type 2 diabetes by reducing the levels of pro-inflammatory cytokines, such as TNF-α, IL-6, and CRP, and regulating the secretion of anti-inflammatory cytokines, such as interleukin-4 (IL-4) and interleukin-10 (IL-10) (Brunkwall and Orho-Melander, 2017; Woldeamlak et al., 2019; Harkins et al., 2020).

T2DM is usually accompanied by chronic low-grade inflammation (Hendijani and Akbari, 2018). Some prospective studies and meta-analyses support the notion that the administration of probiotics could improve glycaemic control (Hu et al., 2017; Wang et al., 2017). However, to our knowledge, the effect of probiotics on inflammatory markers in T2DM patients remains inconsistent. Several RCTs suggest that probiotics reduced the level of chronic systemic inflammatory markers in individuals with T2DM (Tonucci et al., 2017; Raygan et al., 2018; Sabico et al., 2019), while others obtained different conclusions (Feizollahzadeh et al., 2017; Firouzi et al., 2017; Hsieh et al., 2018). No study has simultaneously examined inflammatory biomarkers to capture the interrelation between glycaemic control parameters and inflammatory markers. The aim of the present study is to fill this gap of knowledge.

In the current systematic review and meta-analysis, we aimed to investigate the comprehensive effects of probiotic supplementation on glucose homeostasis and inflammatory markers in adults with T2DM, hoping to obtain more credible evidence-based evidence and provide objective support for the clinical prevention and treatment of T2DM.

## Methods

We conducted the current systematic review and meta-analysis to investigate the comprehensive effects of probiotics on the inflammatory response and glucose homeostasis markers in adults with T2DM according to the PRISMA guidelines (Page et al., 2021a; Page et al., 2021b). This systematic review has been registered in PROSPERO (registration number: CRD42021235998).

### Search Strategy

Two reviewers independently executed a systematic search of randomized controlled trials (RCTs) examining the effect of probiotic supplementation on the inflammatory response and glycaemic control markers in T2DM patients from inception to March 2021 using the following databases: PubMed, Embase, Cochrane Library, and Web of Science. The search strategies used for PubMed, Embase, Cochrane Library, and Web of Science, which were based on the controlled vocabulary terms for each concept (e.g., MeSH) and keyword synonyms, are shown in Table S1. We imposed no language or other restrictions on any of the searches. A manual search was also conducted to identify further relevant studies from the references of included studies and previous systematic reviews.

### Eligibility Criteria

Studies were selected based on the following criteria: (1) studies with participants with T2DM aged ≥18 years old; (2) RCTs; (3) studies in which the intervention was probiotic supplementation; and (4) studies in which changes in inflammatory markers (TNF-α, IL-6, and CRP) between pre- and posttreatment were outcome variables.

The exclusion criteria were as follows: (1) studies with pregnant or breast-breeding individuals and (2) studies with participants who consumed synbiotics, prebiotics, herbs or other supplements (such as micronutrients and other dietary constituents).

### Data Extraction

According to the eligibility criteria, two reviewers independently conducted the literature search and data extraction. The third reviewer resolved any disagreements that occurred between the two data collectors. Details of population characteristics (e.g., sex, age and country), intervention arms (e.g., types of probiotics/placebo, dose, and duration of intervention), study design, and outcomes (e.g., inflammatory markers and glycaemic markers) were extracted from the included studies.

### Risk of Bias of Individual Studies

Two investigators independently evaluated the risk of bias within the randomized controlled studies. The risk of biases, including selection bias, performance bias, detection bias, attrition bias, reporting bias, and other biases, of the included studies was classified as low, high, or unclear using the Cochrane Collaboration tool (Higgins et al., 2011).

### Statistical Analysis

Statistical analysis of the data was conducted using Review Manager 5.3 and STATA/SE 15.1. *p*-values less than 0.05 were regarded as statistically significant. The standardized mean difference (SMD) with 95% confidence interval (CI) was used to analyse the effects of probiotics on glycaemic control and inflammatory markers between the probiotic supplementation and control groups. We used a random-effects model to pool SMDs on glycaemic control and inflammatory markers. When the studies did not provide the standard deviations (SDs), the missing SDs of the mean change were calculated based on the CI, standard error (SE), *p*-value, or correlation coefficient (Corr). The correlation coefficient value was estimated as 0.5 to impute the missing SDs, which were not provided by the authors (Follmann et al., 1992). Heterogeneity among studies was assessed by the Cochrane Q-test. I square (*I* ^2^) was used to quantify the magnitude of heterogeneity. *I* ^2^ values of 25, 50, and 75% represented low, moderate, and high levels of heterogeneity, respectively (Higgins et al., 2003). To explore the possible source of heterogeneity, subgroup analysis and sensitivity analysis were conducted. Subgroup analysis was carried out according to the potentially influential variables, including the duration of interventions (≤8 vs. >8 weeks), doses of probiotic supplementation (<10^10^ vs. ≥10^10^ CFU/day), probiotic strains (single-vs. multistrains), and methods of probiotic administration (food vs. powder or capsule). Sensitivity analysis was conducted by the one-study remove (leave-one-out) approach to assess the pooled SMDs for each glycaemic control and inflammatory marker. In addition, funnel plots and Egger’s test were utilized to evaluate the possible publication bias of the included studies.

## Results

### Search Results and Characteristics of the Included Studies

**FIGURE 2:**
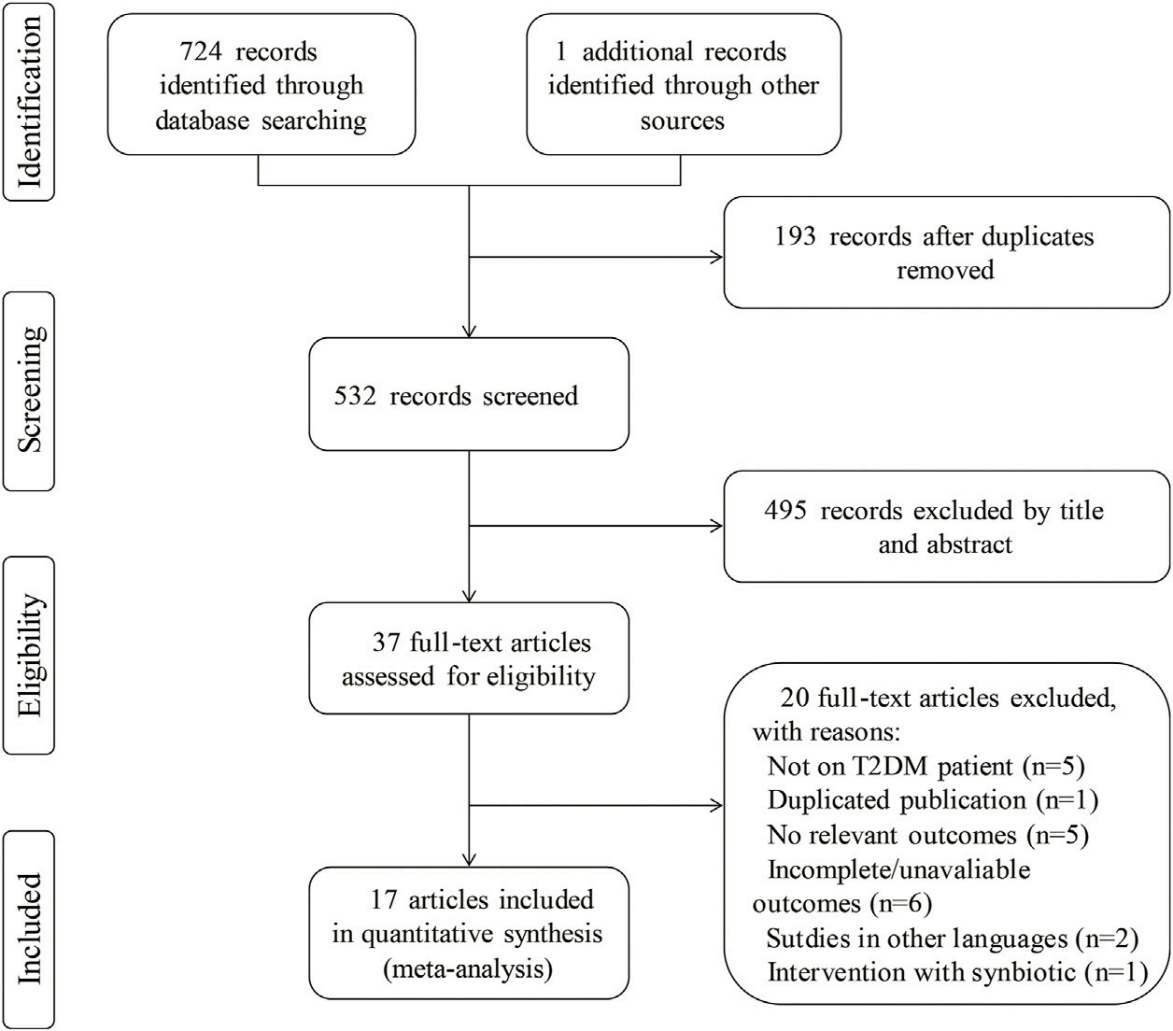
Study flow diagram for study selection.

A total of 725 studies (75 from Cochrane Library, 484 from Embase, 111 from PubMed, 54 from Web of Science and 1 from references) were identified using the search strategies described in the methods section, of which 193 duplicate records were removed. Two investigators independently screened the titles and abstracts and identified 37 articles according to the eligibility criteria described above. Twenty of these articles were excluded according to the inclusion/exclusion criteria. Finally, 17 RCTs were selected for inclusion in the meta-analysis. A flow diagram presents the study selection, and the reasons articles were excluded (Figure 2).

The characteristics of the included studies are summarized in Table 1. Overall, 17 relevant studies were conducted in Europe [Ukraine (Kobyliak et al., 2018a; Kobyliak et al., 2018b), Sweden (Mobini et al., 2017), and Denmark (Hove et al., 2015)], South America [Brazil (Tonucci et al., 2017)], and Asia [China (Hsieh et al., 2018; Fang et al., 2020), Saudi Arabia (Sabico et al., 2019), Malaysia (Firouzi et al., 2017), Iran (Mazloom et al., 2013; Mohamadshahi et al., 2014; Tajadadi-Ebrahimi et al., 2014; Bayat et al., 2016; Feizollahzadeh et al., 2017; Raygan et al., 2018), Turkey (Eliuz Tipici et al., 2020), and Japan (Sato et al., 2017)]. A total of 836 participants from 17 RCTs were randomized into probiotic groups (*n* = 423) and control groups (*n* = 413). Within the 17 RCTs, eight studies used one single-strain probiotic, eight studies used two-strain or multistrain probiotics, and one trial did not report the species of probiotics. Seven studies used food products, such as fermented milk (*n* = 3), yogurt (*n* = 2), soy milk (*n* = 1), and bread (*n* = 1), for probiotic delivery, while others were supplemented in the form of powder (*n* = 5), capsules (*n* = 3), and tablets (*n* = 1). The daily dose of probiotics ranged from 2 × 10^7^ to 1 × 10^12^ colony-forming units (CFU). The median duration of probiotic intervention was 12 weeks, ranging from 4 to 24 weeks.

**TABLE 1:** Characteristics of included studies.

Study	Country	Sample size (P/C)	Ages (year) (P/C)	Underlying disorder	Female (%)	ITT/PP	Study design	Form of probiotics	Probiotics (strain and daily dose)	Duration (weeks)
Tipici et al. (2020)	Turkey	34 (17/17)	30–60	NR	100	ITT	PC	NR	*Lactobacillus GG*, 1 × 10^10^ CFU/day	8
Fang et al. (2020)	China	30 (15/15)	35–68 (57.87 ± 6.31/56.67 ± 7.68)	NR	66.33	PP	DB, PC	powder	*Bifidobacterium animalis A6*, 1 × 10^10^ CFU/day	4
Sabico et al. (2019)	Saudi Arabia	61 (31/30)	30–60 (48.00 ± 8.30/46.60 ± 5.90)	NR	57.38	PP	DB, PC	powder	2 g probiotics mixture (2 × 10^9^ CFU/g, contains *Bifidobacterium bifidum* W23, *Bifidobacterium lactis* W52, *Lactobacillus acidophilus* W37, *Lactobacillus brevis* W63, *Lactobacillus casei* W56, *Lactobacillus salivarius* W24, *Lactococcus lactis* W19, and *Lactobacillus lactis* W58), twice/day	24
Kobyliak et al. (2018a)	Ukraine	58 (30/28)	18–65 (53.40 ± 9.55/57.29 ± 10.45)	NAFLD	NR	ITT	DB, PC	powder	*Lactobacillus* + *Lactococcus* (6 × 10^10^ CFU/g), *Bifidobacterium* (1 × 10^10^ CFU/g), *Propionibacterium* (3 × 10^10^ CFU/g), *Acetobacter* (1 × 10^6^ CFU/g), 10 g/day	8
Kobyliak et al. (2018b)	Ukraine	53 (31/22)	18–75 (52.23 ± 9.69/57.18 ± 9.66)	NR	NR	ITT	DB, PC	powder	*Lactobacillus* + *Lactococcus* (6 × 10^10^ CFU/g), *Bifidobacterium* (1 × 10^10^ CFU/g), *Propionibacterium* (3 × 10^10^ CFU/g), *Acetobacter* (1 × 10^6^ CFU/g), 10 g/day	8
Hsieh et al. (2018)	China	44 (22/22)	25–70 (52.32 ± 10.20/55.77 ± 8.55)	NR	43.18	PP	DB, PC	capsule	*Lactobacillus reuteri* ADR-1, 4 × 10^9^ CFU/day	24
Raygan et al. (2018)	Iran	60 (30/30)	40–85 (60.70 ± 9.40/61.80 ± 9.80)	CHD	NR	ITT	DB, PC	capsule	*Bifidobacterium bifidum* 2 × 10^9^ CFU/day + *Lactobacillus casei* 2 × 10^9^ CFU/day + *Lactobacillus acidophilus* 2 × 10^9^ CFU/day	12
Tonucci et al. (2017)	Brazil	45 (23/22)	35–60 (51.83 ± 6.64/50.95 ± 7.20)	NR	42.22	PP	DB, PC	fermented milk	*Lactobacillus acidophilus* La-5, 1 × 10^9^ CFU/day + *Bifidobacterium animalis subsp. lactis* BB-12, 1 × 10^9^ CFU/day	6
Firouzi et al. (2017)	Malaysia	101 (48/53)	30–70 (52.90 ± 9.20/54.20 ± 8.30)	NR	51.64	PP	DB, PC	powder	*Lactobacillus acidophilus*, *Lactobacillus casei*, *Lactobacillus lactis, Bifidobacterium bifidum, Bifidobacterium longum, Bifidobacterium infantis,* 3 × 10^10^ CFU, twice/day	12
Mobini et al. (2017)	Sweden	29 (14/15)	NR (64.00 ± 6.00/65.00 ± 5.00)	NR	24.14	PP	DB, PC	tablet	*Lactobacillus reuteri* DSM 17938, 1 × 10^10^ CFU/day	12
Feizollahzadeh et al. (2017)	Iran	40 (20/20)	35–68 (56.90 ± 8.09/53.60 ± 7.16)	NR	52.50	PP	DB, PC	soy milk	*Lactobacillus planetarum* A7, 2 × 10^7^ CFU/day	8
Sato et al. (2017)	Japan	68 (34/34)	30–79 (64.00 ± 9.20/65.00 ± 8.30)	NR	27.94	ITT	DB, PC	fermented milk	*Lactobacillus casei strain* Shirota, 4 × 10^10^ CFU/day	16
Bayat et al. (2016)	Iran	40 (20/20)	25–75 (54.10 ± 9.54/46.95 ± 9.34)	NR	70.00	ITT	PC	Yogurt	NR	8
Hove et al. (2015)	Denmark	41 (23/18)	40–70 (58.50 ± 7.70/60.60 ± 5.20)	NR	0	ITT	DB, PC	fermented milk	*Lactobacillus helveticus* Cardi04	12
Tajadadi-Ebrahimi et al. (2014)	Iran	54 (27/27)	35–70 (52.00 ± 7.20/53.40 ± 7.50)	NR	81.48	ITT	DB, PC	bread	*Lactobacillus Sporogenes,* 1 × 10^8^ CFU, 3 times/day	8
Mohamadshahi et al. (2014)	Iran	44 (22/22)	30–60 (53.00 ± 5.90/49.00 ± 7.08)	overweight or obesity	76.19	ITT	DB, PC	yogurt	*Lactobacillus acidophilus* 3.7 × 10^6^ CFU/g, *Bifidobacterium lactic* 3.7 × 10^6^ CFU/g, 300 g/day	8
Mazloom et al. (2013)	Iran	34 (16/18)	25–65 (55.40 ± 8.00/51.80 ± 10.20)	NR	76.47	PP	SB, PC	capsule	*Lactobacillus acidophilus* + *Lactobacillus bulgaricus* + *Lactobacillus bifidum + Lactobacillus casei*	6

CHD, coronary heart disease; P/C, probiotic group/control group; ITT/PP, intention-to-treat/per-protocol; DB, double-blinded; PC, placebo-controlled; SB, single blinded; NR, not reported.

### Risk of Bias in Individual Studies

**FIGURE 3:**
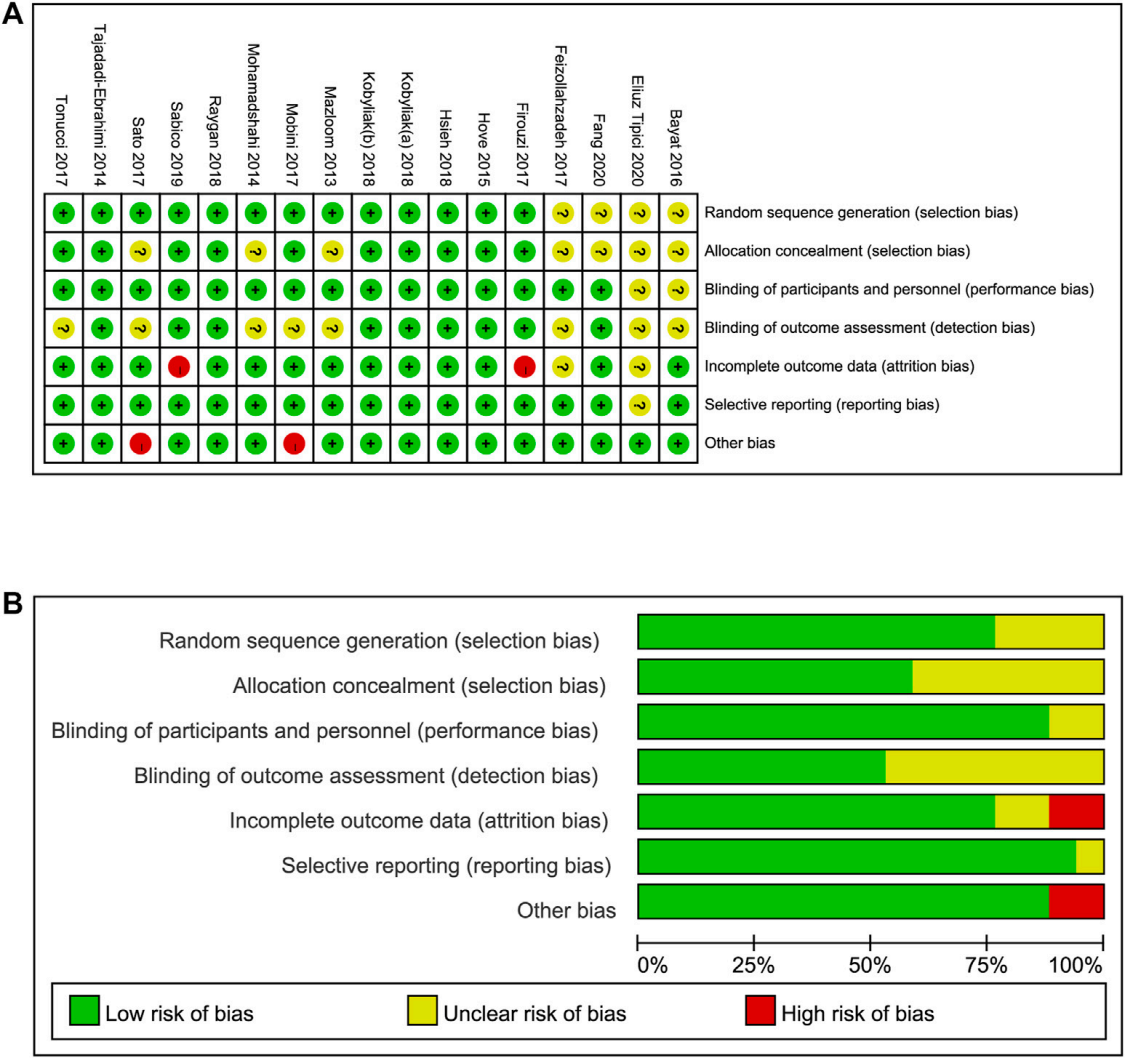
Risk of bias assessment **(A)** details of included studies, **(B)** overall summary.

Two investigators independently assessed the risk of bias of the included studies using the Cochrane collaboration tool. Figure 3 summarizes the risk of bias, including selection bias, attrition bias, performance bias, detection bias, reporting bias, and other biases, among the included studies. Thirteen studies reported adequate random sequence generation, and ten studies described allocation concealment. In 15 studies, the participants and key researchers were blinded. The dropout rates in 13 RCTs were less than 20%. Selective reporting bias was not found. Two studies may have a high risk of other potential bias, according to the funding, which was supported by the probiotic companies. In summary, no significant bias was detected (Figure 3).

### Effects of Probiotics Supplementation on the Inflammatory Response and Glycaemic Control Marker Profile

#### Effects on Pro-Inflammatory Markers (TNF-α, IL-6, and CRP)

##### Effect on TNF-α

**FIGURE 4:**
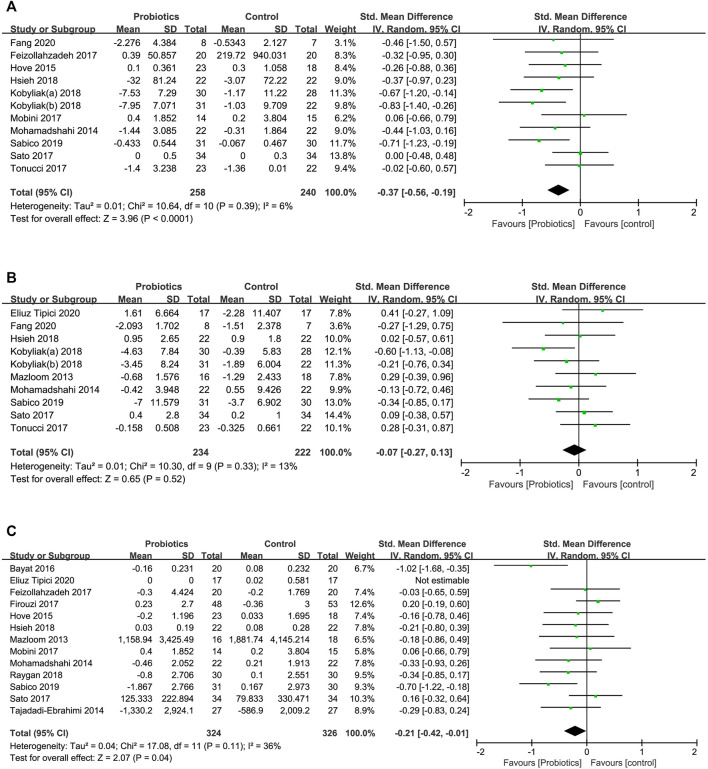
Forest plots of the effects of probiotics on **(A)** TNF-α, **(B)** IL-6, **(C)** CRP.

A total of 11 RCTs assessed the effect of probiotic supplementation on TNF-α and involved 498 individuals (258 consuming probiotics and 240 in the placebo group). The pooled effect of probiotic administration showed a significant decrease in TNF-α levels in comparison with the effects in the control groups (SMD [95% CI]; −0.37 [−0.56, −0.19], *p* < 0.0001), and the interstudy heterogeneity was low (*I* ^2^ = 6%, *p* = 0.390) (Figure 4A).

Subgroup analysis (Table 2) stratified by probiotic dosage, the number of probiotic strains, duration of intervention or method of administration showed that the effects of probiotic supplementation on TNF-α were significantly reduced in studies with the high-dose subgroup (SMD [95% CI]; −0.65 [−0.92, −0.39], *I* ^2^ = 0%), multistrain subgroup (SMD [95% CI]; −0.55 [−0.82, −0.27], *I* ^2^ = 16.6%), short intervention duration subgroup (SMD [95% CI]; −0.47 [−0.72, −0.22], *I* ^2^ = 0%), and administration in the form of powder/capsule subgroup (SMD [95% CI]; −0.56 [−0.81, −0.31], *I* ^2^ = 0%).

**TABLE 2:** Subgroup analysis for the effects of probiotics on TNF-α.

Subgroup	No. of trials	No. of participants	*I* ^2^ (%)	Pooled SMD [95% CI]
Probiotics dose				
≥10^10^ CFU/day	5	231	0	−0.65 [−0.92, −0.39]
<10^10^ CFU/day	5	226	0	−0.12 [−0.38, 0.14]
NR	1	41	0	−0.26 [−0.88, 0.36]
Probiotics strain				
Single	6	237	0	−0.19 [−0.44, 0.07]
Multiple	5	261	16.6	−0.55 [−0.82, −0.27]
Duration of intervention				
≤8 weeks	6	255	0	−0.47 [−0.72, −0.22]
>8 weeks	5	243	19.1	−0.27 [−0.56, 0.01]
Method of administration				
Powder/capsule	6	260	0	−0.56 [−0.81, −0.31]
Food	5	238	0	−0.18 [−0.44, 0.07]

CFU, colony forming unit; SMD, standardized mean difference.

In the sensitivity analysis, we found that the pooled effect of probiotic supplementation on TNF-α was not significantly changed by omitting the studies one at a time sequentially, suggesting that the meta-analysis results were stable and reliable.

##### Effect on IL-6

Ten studies involving 456 patients investigated the effect of probiotic intake and placebo on the serum level of IL-6. The pooled effect of probiotic supplementation indicated no significant effect of probiotics in reducing the serum level of IL-6 compared to the effects in the control group (Figure 4B) (SMD [95% CI]; −0.07 [−0.27, 0.13], *p* = 0.520).

Subgroup analysis showed that there was no significant reduction in IL-6 levels in those subgroups stratified by probiotic dosage, the number of probiotic strains, duration of intervention, or method of administration (Table 3).

**TABLE 3:** Subgroup analysis for the effects of probiotics on IL−6.

Subgroup	No. of trials	No. of participants	*I* ^2^ (%)	Pooled SMD [95% CI]
Probiotics dose				
≥10^10^ CFU/day	6	265	10.4	−0.23 [−0.49, 0.03]
<10^10^ CFU/day	3	157	0	0.13 [−0.19, 0.44]
NR	1	34	0	0.29 [−0.39, 0.96]
Probiotics strain				
Single	4	161	0	0.11 [−0.20, 0.42]
Multiple	6	295	29.0	−0.15 [−0.43, 0.12]
Duration of intervention				
≤8 weeks	7	283	30.7	−0.05 [−0.34, 0.24]
>8 weeks	3	173	0	−0.08 [−0.38, 0.22]
Method of administration				
Powder/capsule	6	265	0.8	−0.22 [−0.47, 0.02]
Food	3	157	0	0.08 [−0.23, 0.40]
NR	1	34	0	0.41 [−0.27, 1.09]

We also performed sensitivity analysis by sequentially eliminating one study at a time. No particular studies significantly affected the pooled effect of probiotic supplementation on IL-6 levels. However, heterogeneity among studies was eliminated (*I* ^2^ = 0%, *p* = 0.960) when the meta-analysis excluded only the trial of Kobyliak et al*.* (2018a).

##### Effect on CRP

The effect of probiotic supplementation on CRP was evaluated in 13 studies including 650 participants. We found a significant effect with a low heterogeneity of probiotic supplementation in reducing serum CRP levels compared to the effects in the control group (SMD [95% CI]; −0.21 [−0.42, −0.01], *I* ^2^ = 36%, *p* = 0.040) (Figure 4C).

In the subgroup analysis, significant beneficial effects of probiotic intervention were observed for CRP in the short intervention duration subgroup (SMD [95% CI]; −0.36 [−0.67, −0.05], *I* ^2^ = 22.9%) (Table 4).

**TABLE 4:** Subgroup analysis for the effects of probiotics on CRP.

Subgroup	No. of trials	No. of participants	*I* ^2^ (%)	Pooled SMD [95% CI]
Probiotics dose				
≥ 10^10^ CFU/day	3	206	74.4	−0.25 [−0.82, 0.31]
< 10^10^ CFU/day	6	295	0	−0.11 [−0.34, 0.12]
NR	3	115	52.9	−0.45 [−1.00, 0.10]
Probiotics strain				
Single	6	276	0	−0.07 [−0.31, 0.17]
Multiple	5	300	50.9	−0.25 [−0.58, 0.09]
NR	1	40	0	-1.02 [-1.68, −0.35]
Duration of intervention				
≤8 weeks	5	212	22.9	−0.36 [−0.67, −0.05]
>8 weeks	7	404	38.8	−0.13 [−0.38, 0.13]
Method of administration				
Powder/capsule	6	329	40.2	−0.19 [−0.48, 0.10]
Food	6	287	41.8	−0.25 [−0.56, 0.06]

In the sensitivity analysis, we found that the heterogeneity among studies did not significantly change when eliminating the study of Bayat et al*.* (Bayat et al., 2016), which decreased the interstudy heterogeneity from 36 to 7%.

In this work, eleven studies reported the effect of probiotic supplementation on TNF-α, ten studies described the effect on IL-6, and 13 studies investigated the effect on CRP and were included in the meta-analysis. Compared to the control group, significant beneficial effects of probiotic supplementation were observed for TNF-α (SMD [95% CI]; −0.37 [−0.56, −0.19], *p* < 0.0001) and CRP (SMD [95% CI]; −0.21 [−0.42, −0.01], *p* = 0.040). However, there was no significant beneficial effect on the IL-6 level (SMD [95% CI]; −0.07 [−0.27, 0.13], *p* = 0.520). In line with our results, a previous meta-analysis also found that these supplementations significantly decreased the levels of TNF-α and CRP but had no effect on IL-6 levels (Tabrizi et al., 2019; Zheng et al., 2019).

#### Effect of Probiotics Supplementation on Glucose Homeostasis (FPG, HbA1c, and HOMA-IR)

##### Effect on FPG

**FIGURE 5:**
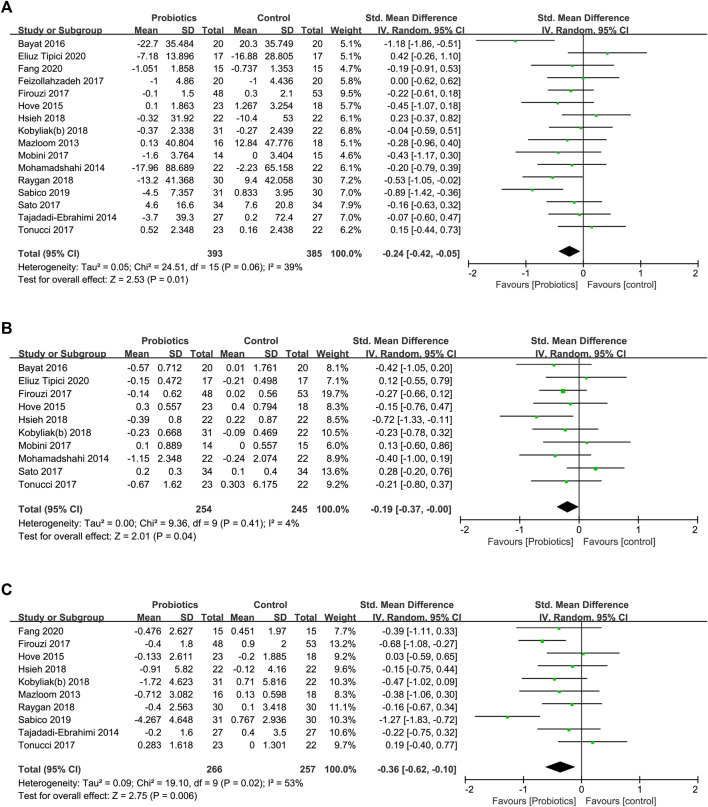
Forest plots of the effects of probiotics on **(A)** FPG, **(B)** HbA1c, **(C)** HOMA-IR.

A total of 778 participants in 16 studies were included in the analysis of the effect of probiotic supplementation on the level of FPG. probiotic supplementation resulted in a significant reduction in FPG levels (SMD [95% CI]; −0.24 [−0.42, −0.05], *p* = 0.010), and the interstudy heterogeneity (*I* ^2^ = 39%, *p* = 0.060) was low (Figure 5A).

Subgroup analysis (Table 5) showed that the effects of probiotic supplementation on FPG were significantly reduced in the multistrain subgroup (SMD [95% CI]; −0.30 [−0.54, −0.05], *I* ^2^ = 33%), long intervention duration subgroup (SMD [95% CI]; −0.34 [−0.60, −0.09], *I* ^2^ = 35.5%), and administration in the form of powder/capsule subgroup (SMD [95% CI]; −0.30 [−0.54, −0.06], *I* ^2^ = 28.9%).

**TABLE 5:** Subgroup analysis for the effects of probiotics on FPG.

Subgroup	No. of trials	No. of participants	*I* ^2^ (%)	Pooled SMD [95% CI]
Probiotics dose				
≥ 10^10^ CFU/day	6	323	49.7	−0.21 [−0.54, 0.11]
< 10^10^ CFU/day	7	340	0	−0.12 [−0.34, 0.09]
NR	3	115	49.1	−0.63 [−1.16, −0.10]
Probiotics strain				
Single	8	340	0	−0.08 [−0.29, 0.14]
Multiple	7	398	33.0	−0.30 [−0.54, −0.05]
NR	1	40	0	−1.18 [−1.86, −0.51]
Duration of intervention				
≤8 weeks	9	374	39.6	−0.14 [−0.41, 0.12]
>8 weeks	7	404	35.5	−0.34 [−0.60, −0.09]
Method of administration				
Powder/capsule	8	412	28.9	−0.30 [−0.54, −0.06]
Food	7	332	43.7	−0.24 [−0.54, 0.05]
NR	1	34	0	0.42 [−0.26, 1.10]

In the leave-one-out sensitivity analysis, the pooled effect of probiotic supplementation on the FPG level remained significant among studies compared to the effects in the control group. Furthermore, after the removal of a single study (Bayat et al., 2016), the interstudy heterogeneity decreased from 39 to 16%, while the FPG reduction was still significant in the probiotic group.

##### Effect on HbA1c

Ten studies described the significant effect of probiotic intake on reducing HbA1c levels in 499 participants (SMD [95% CI]; −0.19 [−0.37, −0.00], *p* = 0.040). The heterogeneity among studies (*I* ^2^ = 4%, *p* = 0.410) was low (Figure 5B).

In the subgroup analysis, significant beneficial effects of probiotic intervention were observed for HbAlc in the multistrain subgroup (SMD [95% CI]; −0.28 [−0.53, −0.02], *I* ^2^ = 0%) and administration in the form of a powder/capsule subgroup (SMD [95% CI]; −0.29 [−0.57, −0.02], *I* ^2^ = 6.6%) (Table 6).

**TABLE 6:** Subgroup analysis for the effects of probiotics on HbAlc.

Subgroup	No. of trials	No. of participants	*I* ^2^ (%)	Pooled SMD [95% CI]
Probiotics dose				
≥ 10^10^ CFU/day	4	232	0	−0.23 [−0.49, 0.03]
< 10^10^ CFU/day	4	186	56.3	−0.12 [−0.57, 0.33]
NR	2	81	0	−0.28 [−0.72, 0.16]
Probiotics strain				
Single	5	216	42.6	−0.06 [−0.42, 0.30]
Multiple	4	243	0	−0.28 [−0.53, −0.02]
NR	1	40	0	−0.42 [−1.05, 0.20]
Duration of intervention				
≤8 weeks	5	216	0	−0.24 [−0.51, 0.03]
>8 weeks	5	283	45.5	−0.14 [−0.47, 0.19]
Method of administration				
Powder/capsule	4	227	6.6	−0.29 [−0.57, −0.02]
Food	5	238	12.0	−0.14 [−0.41, 0.14]
NR	1	34	100.0	0.12 [−0.55, 0.79]

Sensitivity analysis conducted by sequentially omitting one study at a time confirmed that the pooled effect of probiotic supplementation on HbA1c was stable and reliable.

##### Effect on HOMA-IR

Ten studies investigated the effect of probiotic supplementation on the HOMA-IR level in a total of 523 participants. Compared to the effect in the control groups, probiotic supplementation significantly reduced the HOMA-IR level (SMD [95% CI]; −0.36 [−0.62, −0.10], *p* = 0.006), with moderate heterogeneity (*I* ^2^ = 53%, *p* = 0.020) among studies (Figure 5C).

**FIGURE 6:**
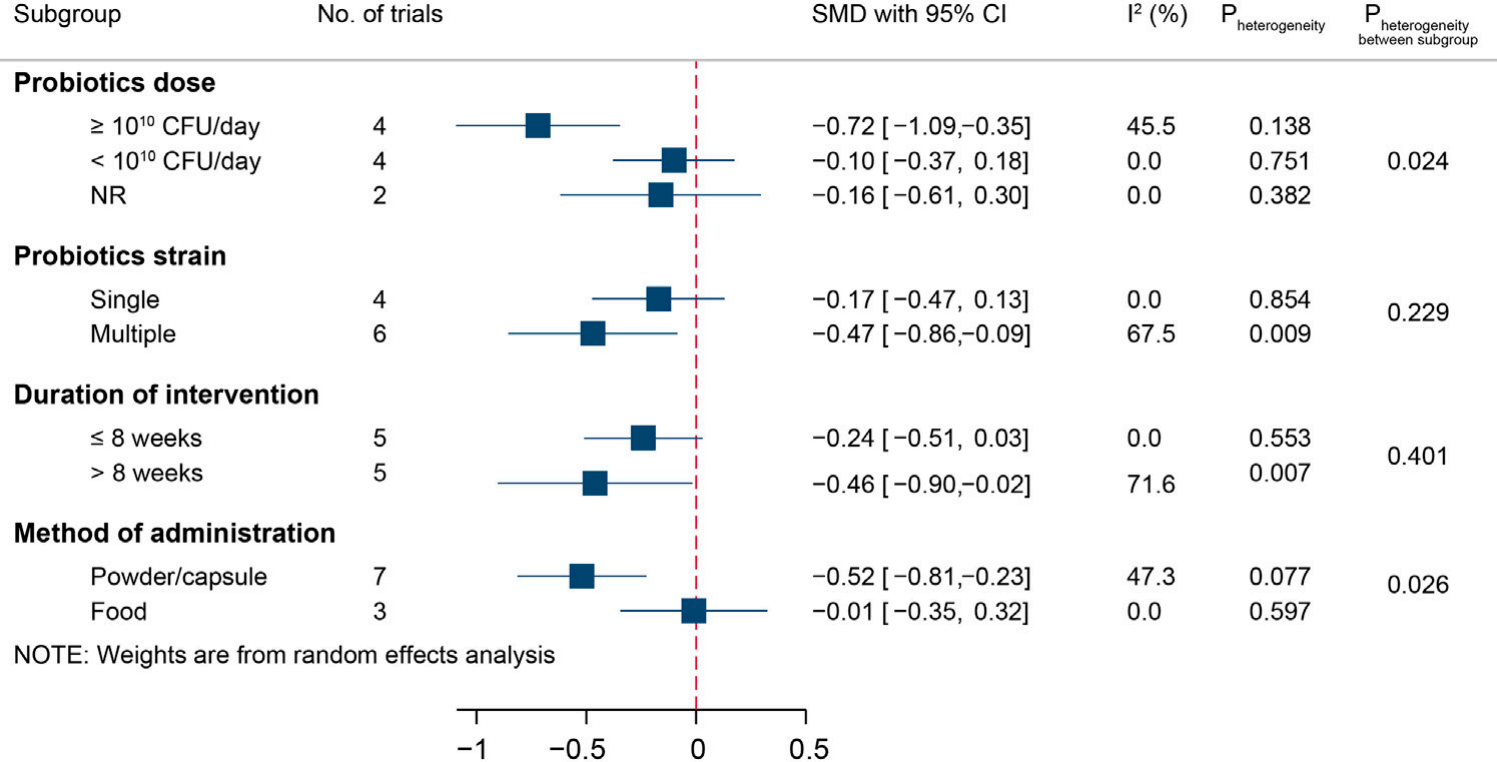
Subgroup analysis for the effects of probiotics on HOMA-IR.

Due to the moderate heterogeneity among studies, we conducted a subgroup analysis for the effect of probiotic administration on HOMA-IR, which is displayed in Figure 6. Probiotic supplementation reduced the HOMA-IR level in the high-dose subgroup (SMD [95% CI]; −0.72 [−1.09, −0.35], *I* ^2^ = 45.5%), multistrain subgroup (SMD [95% CI]; −0.47 [−0.86, −0.09], *I* ^2^ = 67.5%), long intervention duration subgroup (SMD [95% CI]; −0.46 [−0.90, −0.02], *I* ^2^ = 71.6%), and administration in the form of powder/capsule subgroup (SMD [95% CI]; −0.52 [−0.81, −0.23], *I* ^2^ = 47.3%). The interstudy heterogeneity remained similar across all effective subgroups.

Sensitivity analysis confirmed that the HOMA-IR level was not significantly changed compared with that of the control groups. When excluding the study by Sabico et al., we found that the interstudy heterogeneity decreased (*I* ^2^ = 2%, *p* = 0.420), but the effect on HOMA-IR level remained significant (SMD [95% CI]; −0.29 [−0.47, −0.10]).

##### Publication Bias Analysis

Funnel plots and Egger’s test were performed to assess the possible publication bias of the included studies for glycaemic control and inflammatory markers. No evidence of publication bias identified from the funnel plots was found (Figure S1). The results from Egger’s tests also showed no evidence of publication bias for FPG (*p* = 0.990), HbA1c (*p* = 0.927), HOMA-IR (*p* = 0.358), TNF-α (*p* = 0.821), IL-6 (*p* = 0.500), or CRP (*p* = 0.161).

## Discussion

T2DM is associated with long-term chronic low-grade inflammation characterized by an increase in some inflammatory markers, such as TNF-α, IL-6, and CRP, contributing to insulin resistance and the pathogenesis of T2DM (Coope et al., 2016; Eguchi and Nagai, 2017). In recent years, several studies have reported that probiotic supplementation has a variable effect on the inflammatory response in T2DM (Raygan et al., 2018; Anhê et al., 2020; Harkins et al., 2020). Therefore, we conducted the present meta-analysis to evaluate the effect of probiotic supplementation on inflammatory markers and glucose homeostasis in adults with T2DM, attempting to investigate its potential role in modulating gut microbiota to ameliorate glycaemic control and inflammatory markers.

**FIGURE 7:**
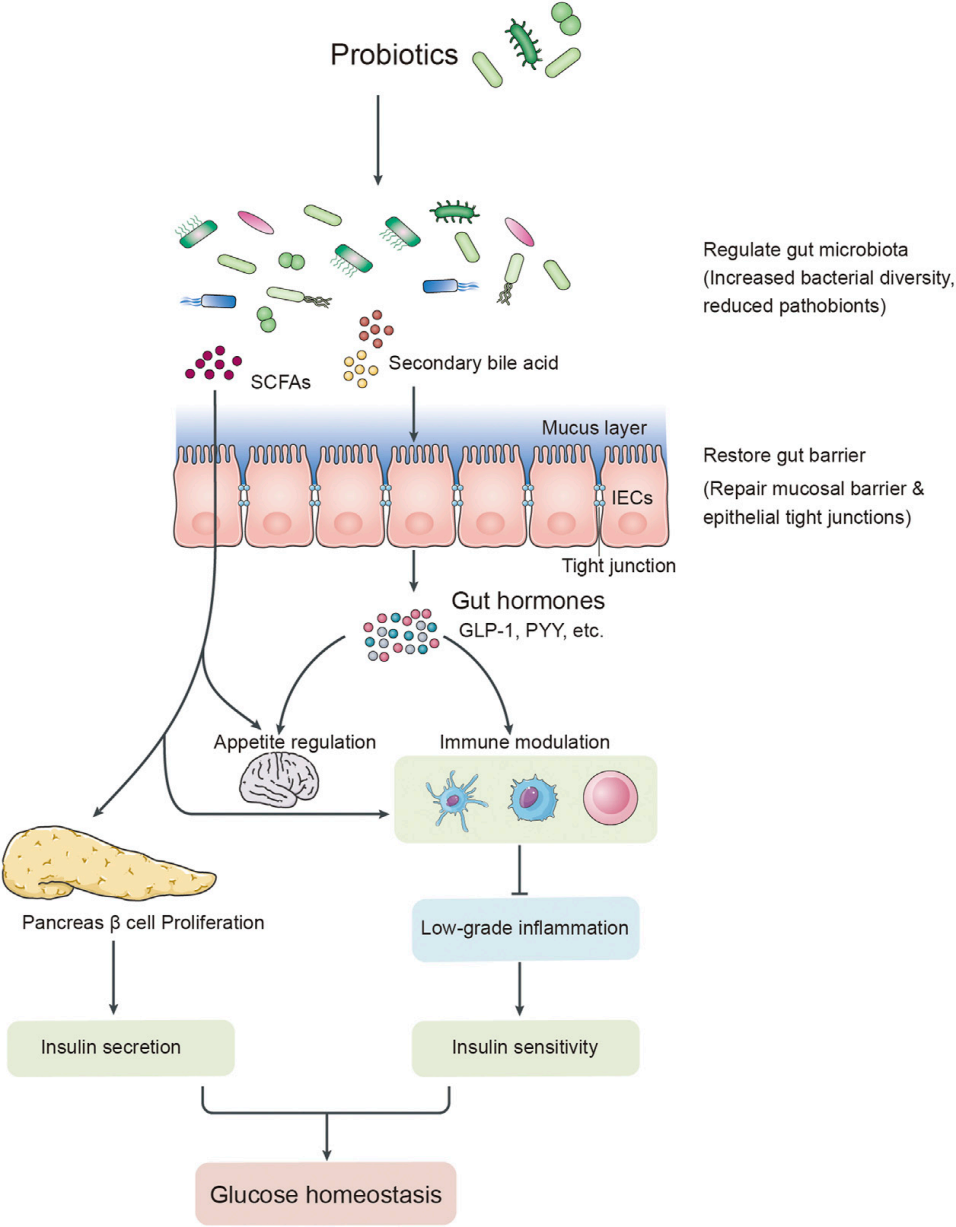
The possible mechanism linking probiotic supplementation and diabetes.

Accumulating evidence provides support for the notion that the gut microbiota is critical to maintain human health (Arora et al., 2021; Wei et al., 2021). Dysbiosis of the gut microbiota could stimulate an increase in the levels of inflammatory markers, such as TNF-α and CRP, leading to aberrant immune activation and chronic low-grade inflammation in individuals with T2DM (Frost et al., 2021; Rinninella et al., 2019). There was also a growing body of evidence suggesting that elevated levels of pro-inflammatory markers, including TNF-α, IL-6, and CRP, play a vital role in the pathogenesis of T2DM (Liaqat et al., 2021; Hyun et al., 2013). These pro-inflammatory markers could inhibit insulin signalling pathways, leading to insulin resistance, dysfunction of β-cells, hyperglycaemia, and the occurrence of T2DM (Liaqat et al., 2021; Harkins et al., 2020; Descamps et al., 2019). However, probiotics play a vital role in glucose homeostasis, insulin resistance, energy metabolism, and chronic low-grade inflammation (Corb Aron et al., 2021; Namazi et al., 2021; Tilg et al., 2020). Some studies have shown that *Akkermansia muciniphila,* as a probiotic, could benefit T2DM by modulating gut microbiota LPS prevention and intestinal permeability reduction (Corb Aron et al., 2021; Zhang et al., 2021). Probiotics could regulate the balance of the host’s gut microbiota by increasing or cross-feeding interactions with other potential beneficial microbes, which could increase insulin secretion, restore insulin sensitivity, and improve glucose homeostasis (Corb Aron et al., 2021; Gurung et al., 2020; Cani et al., 2019). Recent studies have reported that probiotic intake could increase the secretion of hormones, such as GLP-1 and PYY, by regulating the gut microbiota and their metabolites to prevent diabetes (Iorga et al., 2020; Zhang et al., 2020). In addition, probiotics consumed with prebiotics, synbiotics, herbs, micronutrients, or other dietary supplements might enhance the modulation of the gut microbiota and their metabolites, including short-chain fatty acids (SCFAs), neuropeptides, and gastrointestinal peptides (Arora et al., 2021). Moreover, these metabolites could reinforce barrier function, affect gut permeability, suppress chronic low-grade inflammation, and improve insulin resistance and glucose metabolism (Figure 7) (Mulders et al., 2018; Tsai et al., 2019; Gurung et al., 2020; Arora et al., 2021). Some studies also revealed that probiotic supplementation could ameliorate T2DM via inhibition of the expression of pro-inflammatory cytokines, chemokines, or inflammatory proteins (Gomes et al., 2017; Sabico et al., 2019; Tilg et al., 2020).

To obtain accurate results of probiotic supplementation on glycaemic control and inflammatory markers, we excluded studies with participants who consumed prebiotics, synbiotics, herbs, micronutrients, or other dietary supplements, which might influence the gut microbiota or interact with probiotics. Eleven studies reported the effect on TNF-α, ten studies described the effect on IL-6, and thirteen studies investigated the effect on CRP were included in the meta-analysis to evaluate the effects of probiotic supplementation on inflammatory markers. In our study, significant effects of probiotic supplementation were observed on TNF-α and CRP. However, there was no significant effect on IL-6 levels. In line with our results, some studies also showed that the participants consumed probiotics and synbiotics, and these supplementations significantly decreased the levels of TNF-α and CRP but had no effect on IL-6 levels. A previous study found that probiotic and synbiotic administration had a significant effect on CRP levels in individuals with diabetes (Zheng et al., 2019). In addition, a similar result was reported: probiotic and synbiotic supplementation decreased the levels of TNF-α and CRP, but there was no significant effect on IL-6 between the diabetes and control groups (Tabrizi et al., 2019). Subgroup analysis was conducted to assess the potentially influential variables, including the duration of interventions, doses of probiotic supplementation, probiotic strains, and methods of probiotic administration. Significant reductions in TNF-α were detected in consuming multiple strains of probiotics, short intervention duration, high probiotic daily doses, and administration in the form of powder/capsule. There were also significant reductions in CRP levels with short intervention durations and the consumption of multiple strain probiotics. In addition, there were no effects on IL-6 levels among those variables.

These meta-analysis results of glycaemic control parameters were also improved significantly in the levels of FPG, HbA1c, and HOMA-IR compared with the control group. Our results relating to FPG and HOMA-IR were similar to those of previous studies, which showed that probiotic intake could reduce the levels of FPG and HOMA-IR in overweight/obese adults (Wang et al., 2019). However, there was no significant effect on HbA1c levels in their study, which might be because the baseline HbA1c level was lower than that in participants with T2DM. Ardeshirlarijani et al. reported that probiotic intake significantly reduced the level of FPG, while it had no effect on HbA1c levels (Ardeshirlarijani et al., 2019). Moreover, Dong et al. reported that probiotic supplementation improved the levels of HbA1c and HOMA-IR, with no effect on FPG (Dong et al., 2019). In view of previous studies, there were inconsistent results regarding the impact of probiotics on the levels of FPG, HbA1c, and HOMA-IR. Therefore, further evidence is needed to confirm these findings. Subgroup analysis and sensitivity analysis were also conducted in our meta-analysis. The subgroup analysis results showed that the invention groups consuming multiple strains of probiotics or administration in the form of powder/capsule had significantly reduced levels of fasting blood glucose. There were significant reductions in HbA1c levels in those subgroups stratified by probiotic dosage, the number of probiotic strains, duration of intervention, or method of administration. In addition, consistent results were also found for the HOMA-IR level in the subgroup analysis.

However, there were also some potential limitations. First, the sample sizes in all included random clinical trials were small (<60 participants per treatment group). Second, two studies (Hove et al., 2015; Bayat et al., 2016) in our meta-analysis did not provide enough information about interventions. In addition, there were also some differences in intervention types among the included studies, such as the species, doses, survivability of probiotics, and method of probiotic administration, which could also affect the outcomes. These limitations could reduce the reliability of the results from the current meta-analysis. Due to these limitations, some RCTs with well-designed, multicentre, and large sample sizes are still needed for future investigations to provide explicit results.

In summary, probiotic supplementation had a positive effect on glycaemic control markers, including FPG, HbA1c, and HOMA-IR, in parallel with the improvement of some inflammatory markers, such as TNF-α and CRP, in adults with T2DM. This result revealed that the glucose-lowering effect of probiotic supplementation might contribute to its modulation of the gut microbiota, which could exert beneficial effects on glucose homeostasis in patients with T2DM by altering the systemic inflammatory response.

## Conclusion

The evidence supports that probiotic supplementation had beneficial effects on some inflammatory markers (TNF-α and CRP) in parallel with improving glucose homeostasis (FPG, HbA1c, and HOMA-IR) in adults with T2DM. Probiotic supplementation could be beneficial for T2DM patients due to the attenuation of chronic low-grade inflammation by the gut microbiota, which was modulated by probiotics. Therefore, probiotics might become a potential adjuvant therapeutic method for patients with T2DM. However, more well-designed, multicentre RCTs with large sample sizes are needed to further investigate the potential beneficial effects of probiotics in the management of T2DM.

## Data Availability

The original contributions presented in the study are included in the article/Supplementary Material, further inquiries can be directed to the corresponding authors.

## Supplementary Material

Supplementary Table S1 Search strategy.

Supplementary Figure S1 Funnel plots of the effects of probiotics on **(A)** TNF-α, **(B)** IL-6, **(C)** CRP, **(D)** FPG, **(E)** HbA1c, and **(F)** HOMA-IR.

## Abbreviations

CRP, C-reactive protein; FPG, fasting plasma glucose; HbA1c, haemoglobin A1c; HOMA-IR, homeostasis model of assessment of insulin resistance; IL-6, interleukin-6; IECs, intestinal epithelial cells; IRS1, Insulin receptor substrate 1; NF-κB, nuclear factor kappa B; RCT, randomized controlled trial; SCFAs, Short-chain fatty acids; SMD, standardized mean difference; TNF-α, tumour necrosis factor-α; T2DM, type 2 diabetes mellitus.
