# Supplementary figures and images for: Effects of Probiotic Supplementation on Inflammatory Markers and Glucose Homeostasis in Adults With Type 2 Diabetes Mellitus: A Systematic Review and Meta-Analysis
Source: Front Pharmacol. 2021 Dec 10;12:770861. doi: 10.3389/fphar.2021.770861 (PMC8706119; doi:10.3389/fphar.2021.770861)

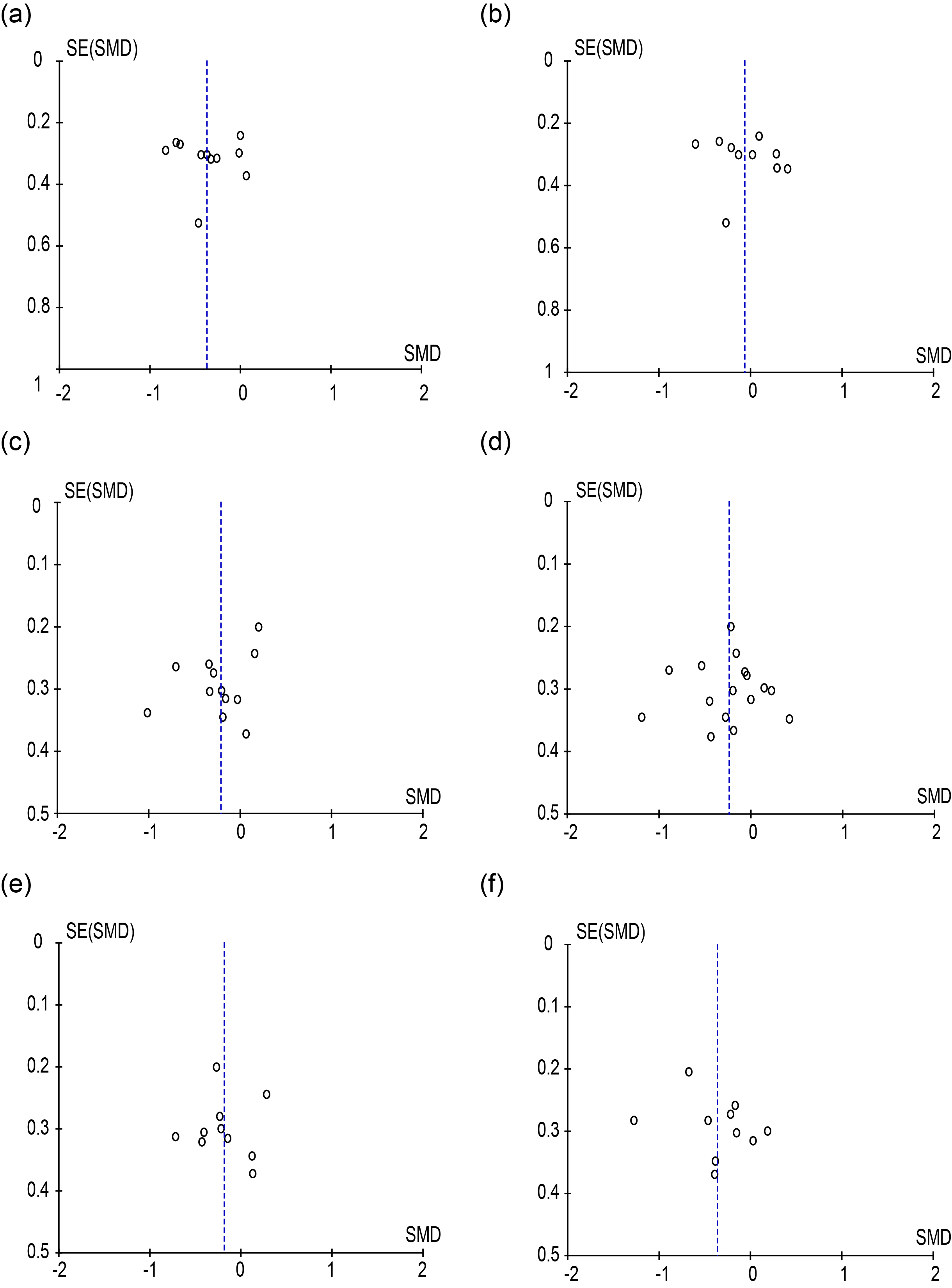

Supplement: Supplementary file 2 [file Image1.JPEG]
